# A multi-country outbreak of *Salmonella* Blockley associated with tomato consumption, July to October 2024

**DOI:** 10.2807/1560-7917.ES.2026.31.40.2500862

**Published:** 2026-10-08

**Authors:** Ann Hoban, Lucy Findlater, Ellen Heinsbroek, Juliette Cavaye, Anais Painset, Clare Maguire, Katie Thorley, Andrew Nelson, Lynda Browning, Derek Brown, Frieda Jorgensen, Jonathan Lighthill, Sandra Simon, Jean-Marc Collard, Nathalie Jourdan-Da Silva, Heather Charles, Jordan Putney, Thai-An Nguyen, Lesley Larkin, Gauri Godbole, Marie Chattaway

**Affiliations:** 1 Gastrointestinal Infections, Food Safety & One Health Division, United Kingdom Health Security Agency (UKHSA), London, United Kingdom; 2 UK Field Epidemiology Training Programme, United Kingdom Health Security Agency (UKHSA), London, United Kingdom; 3 East of England Field Service, United Kingdom Health Security Agency (UKHSA), Cambridge, United Kingdom; 4 Gastrointestinal Bacteria Reference Unit, United Kingdom Health Security Agency, London, United Kingdom; 5 Public Health Wales, Cardiff, United Kingdom; 6 Clinical and Protecting Health Directorate, Public Health Scotland, Glasgow, United Kingdom; 7 Scottish Microbiology Reference Laboratories, Glasgow, United Kingdom; 8 Food, Water and Environmental Laboratory Services, United Kingdom Health Security Agency (UKHSA) Porton Down, Salisbury, United Kingdom; 9 Food Standards Agency, London, United Kingdom; 10 Gastrointestinal Infections, Zoonoses and Tropical Infections Unit/Department of Infectious Diseases, National Reference Centre for *Salmonella* and other Bacterial Enterics, Wernigerode, Germany; 11 Institut Pasteur, Université Paris Cité, Centre National de Référence des *E. coli*, *Shigella* et *Salmonella*, Unité des Bactéries pathogènes entériques, Paris, France; 12 Santé publique France, Saint-Maurice, France; 13 Foods Standards Scotland, Aberdeen, United Kingdom; 14 Applied Science, Research & Technology, Inc., Smyrna, Georgia, United States; 15 Division of Foodborne Waterborne and Environmental Diseases, Centers for Disease Control and Prevention, Atlanta, Georgia, United States; 16 National Institute for Health Research, Health Protection Research Unit in Gastrointestinal Infections, University of East Anglia, Norwich, United Kingdom; 17 National Institute for Health Research, Health Protection Research Unit in Public Health Genomics, University of Birmingham, Birmingham, United Kingdom

**Keywords:** Salmonella outbreak, Blockley, tomatoes, genomics, epidemiology

## Abstract

Between July and October 2024, 216 cases with genetically related *Salmonella* Blockley sequence type 52 were identified in France (n = 56), Germany (n = 30), the United Kingdom (UK) (n = 129) and the United States (US) (n = 1). Case interviews in the UK identified tomatoes as a possible vehicle of infection. A rapid case–control study with 29 cases and 87 controls was conducted to investigate this hypothesis, using pre-existing control data. Multivariable, Firth penalised logistic regression was used to explore associations between illness, food exposures and retailers. Consumption of tomatoes and shopping at Retailer A (adjusted odds ratio (aOR) = 10.75; 95% confidence interval (CI): 1.52–103.16) was associated with being a case. *Salmonella* spp. was not detected in any of the 312 tomato samples tested by a standard culture method, but a low concentration of *Salmonella* DNA was detected in two enriched broths by PCR. These detections could not be confirmed by culture. Evidence derived from epidemiological, microbiological and food chain investigations in the UK identified imported tomatoes from Morocco as the most likely vehicle of infection.

**Key public health message**

**What did you want to address in this study and why?**

*Salmonella* is a bacterium that can cause diarrhoea, fever and stomach cramps and in some cases can lead to severe illness requiring hospital treatment. In summer 2024, an unusual increase in infections caused by a rare type of *Salmonella* called *Salmonella* Blockley was identified. We investigated the outbreak to identify the cause of these infections and help prevent further cases.

**What have we learnt from this study?**

When information obtained from patient interviews, food testing and supply chain investigations was considered together, tomatoes were identified as the most likely source of the outbreak.

**What are the implications of your findings for public health?**

Fresh produce can cause widespread outbreaks when contamination occurs within international food supply chains. Early detection, rapid information sharing and collaboration between public health and food safety agencies are important for reducing the impact of these outbreaks.

## Background

Non-typhoidal *Salmonella* spp. (NTS) is a globally important pathogen, with estimates suggesting over 93 million cases of gastroenteritis occurring globally each year [1]. Typical symptoms include diarrhoea, nausea, vomiting, fever and abdominal pain. While most infections are self-limiting, young children, adults aged ≥ 65 years and individuals with weakened immune systems are more prone to developing a severe disease. Most NTS infections are considered food-borne, and outbreaks have been linked to a wide range of food products including meat, eggs, fresh produce and ready-to-eat items [2-5].

In the United Kingdom (UK), *Salmonella* spp. surveillance in humans relies on the statutory reporting of laboratory-confirmed infections, with patient isolates routinely referred to the relevant National Reference Laboratories (NRL) for confirmation and typing. Since 2014, the UK Health Security Agency (UKHSA, formerly Public Health England) and the Scottish Microbiology Reference Laboratory (SmiRL) NRLs have implemented whole genome sequencing (WGS) as the primary method for *Salmonella* characterisation, replacing traditional serotyping and phage typing. The adoption of WGS has substantially improved the detection and characterisation of outbreaks enabling rapid identification of genetically related cases and supporting earlier investigation of potential food-borne sources [6].

## Outbreak detection

On 20 August 2024, the UKHSA NRL for *Salmonella* noted that a rare non-typhoidal serovar *Salmonella* Blockley (antigenic profile: 6,8:k:1,5) had been isolated from 35 cases across England and Wales. Since 2014, laboratory diagnoses of *S.* Blockley reported to the UKHSA national surveillance have ranged from 0 to 6 cases per month, with the majority considered travel-associated. This increase in submissions was also observed in Scotland.

Subsequent WGS analysis identified submitted isolates of *S.* Blockley had a specific genetic profile (5-single nucleotide polymorphism (SNP) address: 1.1.2.2.39.57.%). A multi-agency incident management team (IMT) was convened upon notification of an increase in reporting as per established protocols [7]. Information available at the time indicated cases within this cluster had not travelled and were resident in multiple regions of the UK, suggestive of a nationally distributed food vehicle. Here we describe the outbreak investigation undertaken with the aim of identifying the outbreak vehicle and the source of the outbreak to implement control measures.

## Methods

### Surveillance of salmonellosis in the United Kingdom

Laboratory diagnoses of salmonellosis in the UK are dependent on symptomatic cases presenting to healthcare services and a sample being taken for analysis. In England and Wales, *Salmonella* isolates identified by diagnostic testing laboratories are routinely submitted to the UKHSA NRL for *Salmonella* for confirmation and further characterisation with WGS. In Scotland, *Salmonella* isolates are referred to the SmiRL for WGS. Hierarchical single linkage clustering is performed on the pairwise SNP difference between all isolates within an eBurst group (eBG) at various distance thresholds to generate a SNP address, as previously described [8,9]. Antimicrobial resistance determinants are analysed using a customised algorithm Genefinder V1–5.

Upon notification of laboratory-confirmed salmonellosis to public health authorities, cases may be followed up by local Environmental Health Officers (EHOs). The extent and content of follow-up questionnaires vary between local authorities and UK nations. While some questionnaires include questions about salad consumption, they do not typically collect detailed information on specific salad ingredients or individual salad items, limiting the systematic collection of this exposure information.

### Outbreak case definition

We created a definition for confirmed cases of this outbreak based on the criteria outlined (Box).

Box **Definition of confirmed case of *Salmonella* Blockley in a multi-country outbreak, 2024**

**Epidemiological criteria:**

• A person resident in France, Germany, the United Kingdom or the United States.

**Laboratory criteria:**

• A specimen tested positive for *S*. Blockley sequence type (ST) 52 between 1 August and 31 October 2024

AND

• *S*. Blockley isolate belonged to the same 5-SNP single linkage cluster

OR

• *S*. Blockley isolate clustered within five allelic differences of another confirmed outbreak isolate by cgMLST

OR

• *S*. Blockley isolate HC5_385614 by the EnteroBase HierCC scheme [10,24].

cgMLST: core-genome multi-locus sequence typing; SNP: single nucleotide polymorphism.

### Epidemiological investigations

All confirmed cases in England and Wales meeting the case definition were identified through the review of routine WGS data in the laboratory reporting system UKHSA Gastro Data Warehouse (GDW). For cases in Scotland, WGS data at SmiRL were reviewed. An alert was disseminated internationally via the European Centre for Disease Prevention and Control (ECDC) Food and Waterborne Diseases (FWD) platform EpiPulse on 4 September 2024 (2024-FWD-00076). Accession numbers for representative outbreak isolates were communicated via Epipulse and uploaded to Enterobase (https://enterobase.warwick.ac.uk/) [10].

### Analytical studies

An epidemiological investigation cell was convened under the IMT to lead on the design of a case–control study. The epidemiological investigation cell contacted the confirmed cases for a telephone interview. Confirmed cases resident in the UK were included in the case–control study if they had no history of foreign travel and no close contact with other individuals with diarrhoea in the 7 days before onset of illness. We created an outbreak questionnaire which was used in the interviews and collected information on demographics, symptoms, close contacts, travel and day trips, animal contact and food consumption (including food exposures outside home, i.e. in restaurants and cafes) in the 7 days prior to symptom onset. The full questionnaire is available in Supplement.

Controls were recruited 20–21 November 2023 via a market research panel for a previous *Salmonella* outbreak. Controls were UK residents with no history of diarrhoea or vomiting in the previous week. To match more closely the age and regional distribution with the confirmed cases, controls from Northern Ireland or aged under 10 years were excluded. This was due to a lack of cases reported in Northern Ireland and a low number of young children reported at the time of study design. Controls completed an electronic questionnaire on demographics, travel history, and fresh produce and egg consumption in the 7 days before.

To compare the distribution of demographic variables between cases and controls, the Mann–Whitney U test was used for continuous variables (age) and Pearson’s chi-square test for categorical variables (age group, sex). To compare the region of residence between cases and controls, Pearson’s chi-square test with Monte Carlo simulation was used due to small numbers. This approach approximates the p value by repeatedly simulating contingency tables under the null hypothesis and estimating the proportion of simulated test statistics at least as large as the observed value (10,000 replicates).

Odds ratios (OR) and 95% confidence intervals (CI) were calculated using Firth penalised logistic regression to estimate associations between case or control status and specific food exposures [11]. Firth penalised logistic regression was chosen a priori to improve robustness of OR estimates for exposure strata with small numbers. A multivariable logistic regression model was built, including age, sex and region as a priori confounders. Food exposures associated with an OR > 1.0, a p value < 0.2, and where at least 20% of cases were exposed, were included in the multivariable model. Subsequently, food exposures which appeared protective against illness, with an OR < 1.0 in the multivariable model, were removed from the model. Data were analysed with R version 4.2.1 (https://www.r-project.org/).

### Food chain investigations in the United Kingdom

Food chain investigations were led by the UK Food Standards Agency (FSA). The FSA used the exposure information derived from case interviews to inform supply chain investigation. Information required for food chain traceback analysis was requested from the food business operators (FBO), including their Hazard Analysis and Critical Control Point (HACCP) plan, source of the raw materials, food safety management system information for the site along with details of any *Salmonella* spp. detections from the FBO’s own internal testing undertaken as part of the company’s food safety management system.

### Microbiological analyses of food samples

Due to several cases reporting consumption of tomatoes, environmental health officers from local authorities or UKHSA staff collected samples of packaged tomatoes from retail outlets in the UK. These samples were transported to UKHSA Food, Water and Environmental Microbiology Service (FWEMS) laboratories for testing. From each pack, 25 g of tomatoes as well as a rinse (using buffered peptone water (BPW)) of the entire remaining tomatoes were tested for the presence of *Salmonella* based on ISO 6579–1:2017 [12]. In addition to the ISO 6579-1:2017 culture method, the BPW enrichment broths were tested for *Salmonella* by a real-time PCR using a RapidFinder *Salmonella* Species, Typhimurium and Enteritidis Multiplex PCR Kit (Thermo Fisher Scientific, San Diego, the United States (US)) according to the manufacturers’ instructions, where 10 μL of the enriched broth and 10 μL of proteinase K were added to a lysis tube, then, following a thermal lysis, 20 μL of the upper layer was added to the PCR tube containing lyophilised reagents.

### Characterisation of isolates

Due to the rarity of *S*. Blockley ST52, we analysed all publicly available *S*. Blockley sequences with core genome multilocus sequence typing (cgMLST) [6,7] and SNP-based methods [5,9]. Sequencing data from the National Center for Biotechnology Information (NCBI) and Enterobase were downloaded. A maximum likelihood phylogenetic tree was constructed using alignments of SNP position extracted from SnapperDB and IQtree [13].

## Results

### Descriptive epidemiology

By 31 October 2024, 216 confirmed cases were identified (Figure 1). In the UK, 129 confirmed cases were detected in England (n = 79), Scotland (n = 33) and Wales (n = 17). No cases were identified in Northern Ireland. Matching cases in France were detected via Enterobase on 21 August 2024. After the notification on EpiPulse on 4 September 2024, France, Germany and the US informed about their confirmed or probable cases. In total, matching *S*. Blockley cases were identified in two EU countries (France: 56 cases; Germany: 30 cases) and in the US (one case), although the US case reported travel to the UK before symptom onset.

**Figure 1:**
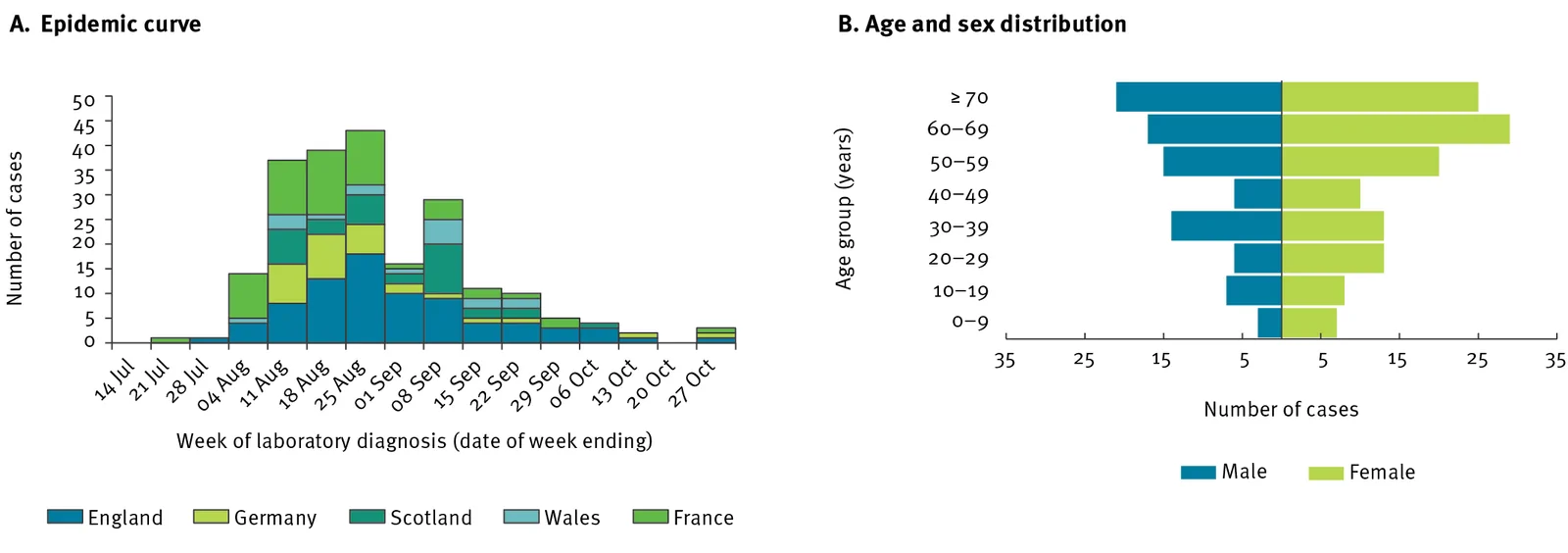
Epidemic curve and age and sex distribution of confirmed cases with *Salmonella* Blockley infection in a multi-country outbreak, France, Germany, the United Kingdom, July–October 2024 (n = 215)

Panel A. Date of sampling is given for cases in France and the United Kingdom; date of notification for the cases in Germany.

Panel B. One case with missing age.

Sampling dates in France, Germany and the UK ranged from 20 July to 21 October 2024 (Figure 1). Cases were aged from < 1 years to 88 years: the most affected age groups were those aged ≥ 60 years (60–69 years: n = 46; 21% and ≥ 70 years: n = 46; 21%). The median age was 55.5 years. More cases were females (125/215; 58%) than males (90/215, 42%).

Cases within the UK were unevenly distributed across the country (Figure 2). The highest incidences were reported in Scotland (33 cases; 0.61/100,000 population), Wales (17 cases, 0.54/100,000 population) and East of England region (29 cases; 0.45/100,000 population). Information on hospitalisation was available for 45 UK cases; 21 cases were admitted to hospital.

**Figure 2:**
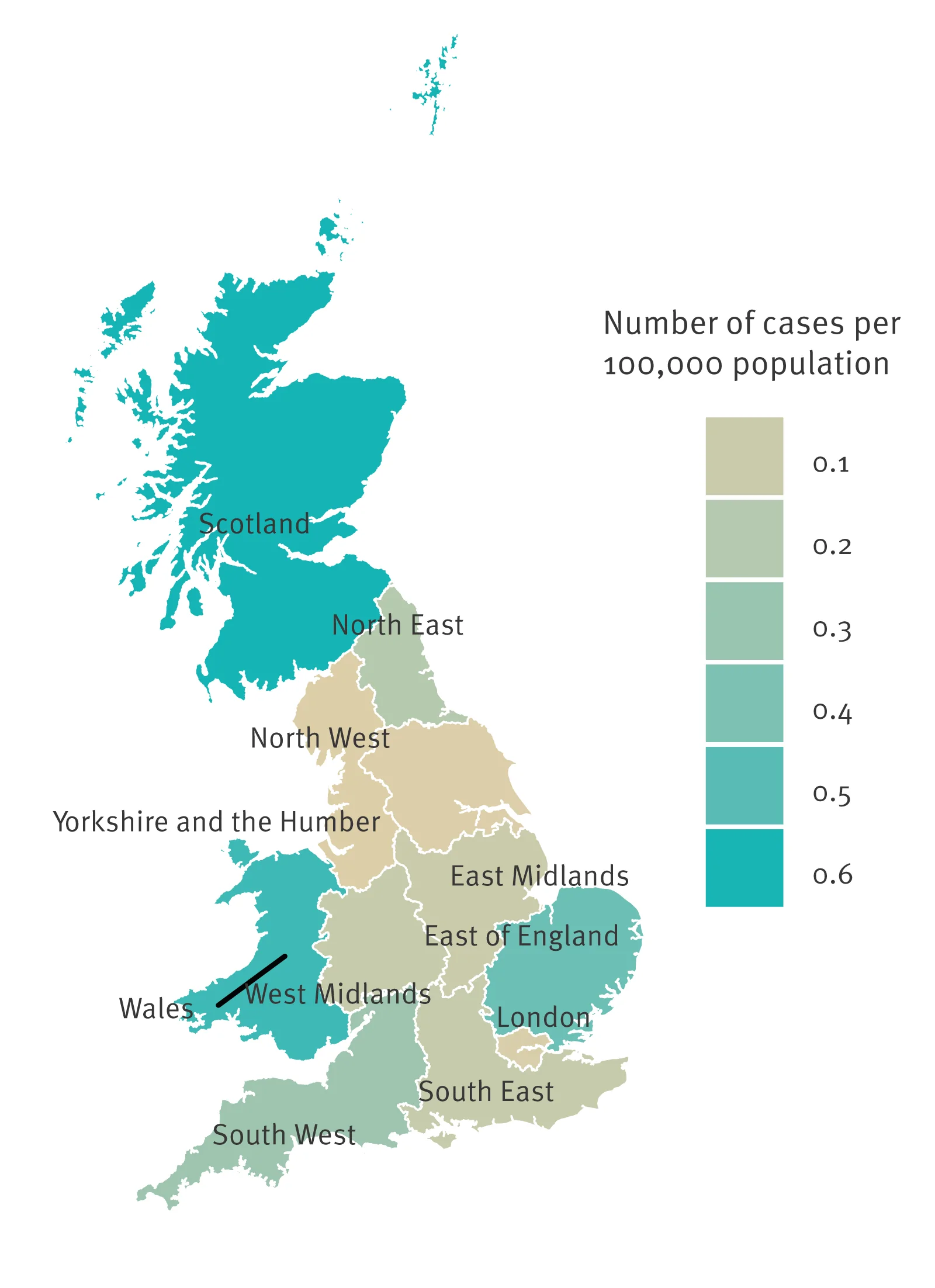
Number of cases with *Salmonella* Blockley infection per 100,000 population, United Kingdom, 2024 (n = 129)

Source: NUTS1 boundaries from data.gov.uk/ONS

### Case interviews

For hypothesis generation, we had data from local questionnaires of 10 UK cases with symptom onsets between 25 July and 15 August 2024. Only one case travelled abroad in the week before symptom onset, therefore their infection was suggestive of domestic acquisition. Tomatoes were voluntarily mentioned in three questionnaires, and eight cases reported shopping at Retailer A. Information shared between public health agencies and reference laboratories across countries revealed that cases in the other affected countries had also consumed tomatoes.

### Analytical epidemiological investigations in the United Kingdom

Within the UK, outbreak questionnaires were completed with 29 cases whose disease onset dates were between 23 July and 26 August 2024. A high proportion of cases reported consumption of tomatoes (27/29 cases) within 7 days before falling ill. Other foods frequently reported by these cases included cucumber (n = 20), salad leaf (n = 18), poultry (n = 24), any dairy milk (n = 24), cheddar cheese (n = 19) and eggs (n = 17). Three cases were unlikely to have eaten meat, as one case followed a no/very low protein diet and two followed a mostly vegetarian diet.

Overall, 29 cases and 86 controls were included in the case–control study. Cases and controls had a very similar sex distribution (62% vs 63% male) and age distribution (median age 57 years vs 55 years (Table 1). Differences were observed in geographical distribution, with more cases living in the East of England and more controls living in the South East and London regions.

**Table 1:** Characteristics of cases (n = 29) and controls (n = 86) in an investigation of an outbreak of *Salmonella* Blockley, United Kingdom, 2024

Characteristics	Cases (n = 29)		Controls (n = 86)	
	n	%	n	%
Sex (p = 1.00)				
Female	18	62	54	63
Male	11	38	32	37
Age (years) (p = 0.685)				
Median	57		55	
Lower quartile	36		42	
Upper quartile	61		66	
Minimum	11		20	
Maximum	82		81	
Age group (years) (p = 0.109)				
10–19	2	6.9	0	0
20–29	2	6.9	7	8.1
30–39	4	14	9	10
40–49	2	6.9	18	21
50–59	10	34	20	23
60–69	6	21	17	20
≥ 70	3	10	15	17
Region (p = 0.009)				
East Midlands	1	3.4	7	8.1
East of England	9	31	10	12
London	0	0	12	14
North East	1	3.4	2	2.3
North West	0	0	8	9.3
Scotland	4	14	3	3.5
South East	2	6.9	13	15
South West	4	14	10	12
Wales	4	14	5	5.8
West Midlands	4	14	7	8.1
Yorkshire and Humber	0	0	9	10

To compare differences in the distribution of demographic variables between cases and controls, we used Pearson’s chi-square test for categorical variables, with Monte Carlo simulation for the region variable due to low numbers of observations expected per stratum. The Mann–Whitney U test was used for continuous variables.

Tomatoes were the food item most strongly associated with illness in univariable analysis (unadjusted OR (uOR) = 7.23; 95% confidence interval (CI): 2.17–37.2), followed by strawberries (uOR = 4.7; 95% CI: 1.8–12.4) and orange peppers (uOR = 3.8; 95% CI: 1.4–10.7) (Table 2). In the multivariable model, when adjusted for demographic variables and the consumption of other food, no exposures were significant. However, tomato consumption had the highest adjusted OR (aOR) (aOR = 3.80; 95% CI: 0.96–21.30; p value = 0.058).

**Table 2:** Number and percentage of cases (n = 29) and controls (n = 86) and odds ratios in univariable and multivariable regression models in an investigation of an outbreak of *Salmonella* Blockley, United Kingdom, 2024

Food item	Cases (n = 29)		Controls (n = 86)		Univariable model			Multivariable model		
	n	%	n	%	Unadjusted OR	95% CI	p value	Adjusted OR	95% CI	p value
Tomatoes	27	93	52	60	7.23	2.17–37.2	< 0.001	3.80	0.96–21.30	0.058
Strawberries	12	41	11	13	4.69	1.81–12.4	0.002	1.96	0.50–8.13	0.330
Orange peppers	9	31	9	10	3.78	1.35–10.7	0.012	1.79	0.31–10.97	0.516
Yellow peppers	13	45	22	26	2.35	0.98–5.60	0.055	1.39	0.29–6.52	0.669
Cucumber	20	69	42	49	2.26	0.96–5.63	0.062	NA		
Mixed salad leaf	10	34	17	20	2.14	0.84–5.32	0.11	1.37	0.36–5.15	0.634
Green peppers	9	31	16	19	1.98	0.76–5.02	0.2	1.03	0.25–3.86	0.970
Blueberries	9	31	17	20	1.84	0.71–4.63	0.2	1.05	0.27–3.91	0.944
Iceberg lettuce	13	45	28	33	1.68	0.71–3.93	0.2	NA		

CI: confidence interval; NA: not analysed; OR: odds ratio.

Odds ratios were calculated using Firth penalised logistic regression [11]. In multivariable model, ORs were adjusted for food items with OR > 1 in the univariable model, and for sex, region and age (age as a restricted cubic spline to allow for nonlinearity).

When we combined consumption of tomatoes with supermarket chain (Retailer A, Retailer B, both, or neither supermarket), eating tomatoes and shopping at Retailer A had higher odds (aOR = 10.75; 95% CI: 1.52–103.16) of being a case than eating tomatoes purchased at other supermarkets, or not eating tomatoes, when adjusted for other food items and demographics (Table 3).

**Table 3:** Association with food exposure and retailer used for shopping groceries in a multivariable regression model in an investigation of an outbreak of *Salmonella* Blockley, United Kingdom, 2024^a^

Exposure	Adjusted OR	95% CI	p value
Tomato consumption and retailer used for shopping groceries			
Retailer A only	10.75	1.52–103.16	0.017
Retailer A and B	4.13	0.68–31.60	0.126
Retailer B only	2.55	0.42–18.38	0.312
Did not shop at Retailer A or B	3.29	0.53–25.50	0.203
No tomato consumed	1.0	NA	
Other food items			
Strawberries	2.01	0.50–9.16	0.327
Yellow peppers	1.66	0.37–7.34	0.496
Mixed salad leaf	1.34	0.36–4.87	0.654
Iceberg lettuce	1.19	0.36–3.86	0.768
Orange peppers	1.18	0.17–8.06	0.863
Blueberries	1.14	0.28–4.56	0.848
Cucumber	1.01	0.27–3.82	0.988

CI: confidence interval; NA: not analysed; OR: odds ratio.

^a^ Results from a case–control study with 29 cases and 86 controls.

### Food chain investigations in the United Kingdom

Food chain investigations identified that UK Retailer A received tomatoes from three countries: Morocco, the Netherlands and the UK. Between July and September 2024, Retailer A stores in Scotland and East of England were supplied with tomatoes from Grower X in Morocco.

### Sampling and testing of tomatoes

Samples of tomatoes (n = 312) from four national UK retailers were tested for *Salmonella* spp. The use-by-dates recorded for samples were in September and October 2024, with the earliest samples examined on 19 September. Of the 312 samples, 185 (59.3%) were from Morocco, 63 (20.2%) from the Netherlands, 40 (12.8%) from the UK and 4 (1.3%) from Spain. For 20 (6.4%) samples, the country of origin was not known. The sampled varieties included baby plum, beef, cherry, classic salad and tomatoes on the vine. All samples tested negative for *Salmonella* spp. by the ISO method.

With PCR, *Salmonella* DNA was detected at a low concentration (quantification cycle (Cq): ca 34–35) in enrichment broths of samples from two unopened packages of cherry tomatoes from Grower X purchased at Retailer A: one with a use-by-date of 27 September 2024 and the other with a use-by-date of 14 October 2024. However, we could not detect *Salmonella* in the enrichment broths by culture. Detection of DNA in the absence of a culture confirmed *Salmonella* result may be due to detection of dead cells, competition from other bacteria resulting in failure of culture-confirmation, and/or free DNA in the sample.

### Characterisation of *Salmonella* Blockley isolates

The outbreak strain was *S*. Blockley eBG 151, ST52, HC5_385614, UKHSA SNP address 1.1.2.2.39.57.%. Genomic analysis suggested antimicrobial resistance to aminoglycosides *(aac(6’)-ly*) and one mutation indicated potential reduced susceptibility to fluoroquinolones (*parC* [57:T-S]).

There were 1,046 *S*. Blockley ST52 isolates in Enterobase up to October 2024, a cgMLST tree of these isolates demonstrates substantial diversity (Figure 3). Of 578 isolates with a known source, 466 were from humans, 89 were from poultry, 11 were from swine, 11 were bovine, 4 were from reptiles and 3 were avian. The main hierarchical clusters at the single linkage clustering at the 5 allelic level (HC5) were HC5_71554 (n = 215), which was from a mixture of human and poultry sources, HC5_385614 (n = 136), which was from humans only, and HC5_1345 (n = 175) which was from humans and was associated with travel to Morocco.

**Figure 3:**
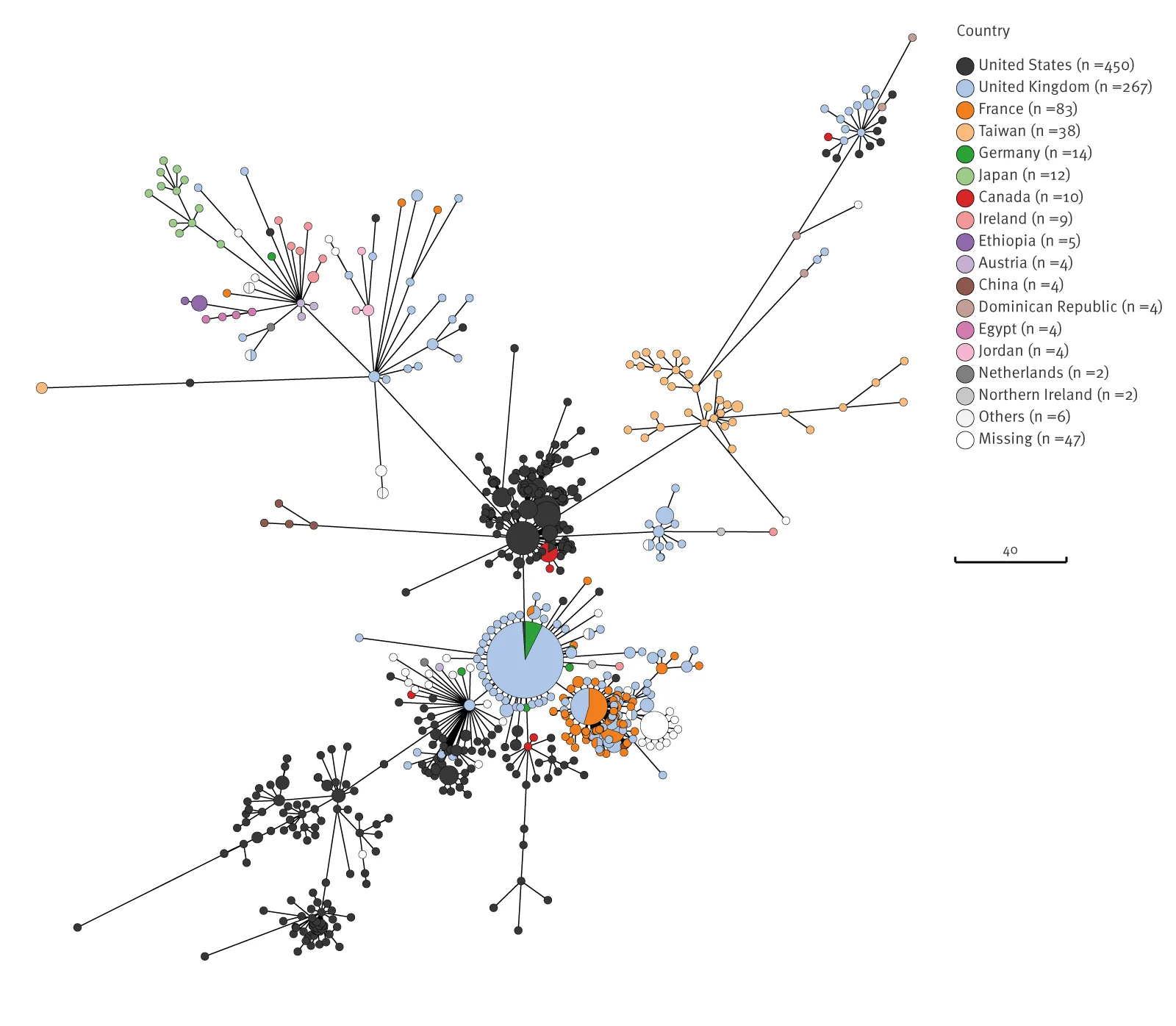
Visualisation of the genomic clustering using core genome multilocus sequence typing of *Salmonella* Blockley isolates (ST52) available on EnteroBase, by country, 1955–October 2024 (n = 1,046)

cgMLST: core genome multilocus sequence typing; ST: sequence type.

There are 446 human isolates, 119 from animals, 13 from the environment and the others are from an unknown source. Annotation highlights the main HC5 subgroups with non-human sources noted within a cluster.

Hierarchical clustering showed the isolates from confirmed cases reported in France, Germany, US and the UK demonstrated limited genetic diversity within the current outbreak cluster and belonged to HC5_385614 (Figure 4). Within the t5.57 outbreak cluster in the UK, the maximum SNP distance was 7 SNPs, and the average was 0.83 SNPs.

**Figure 4:**
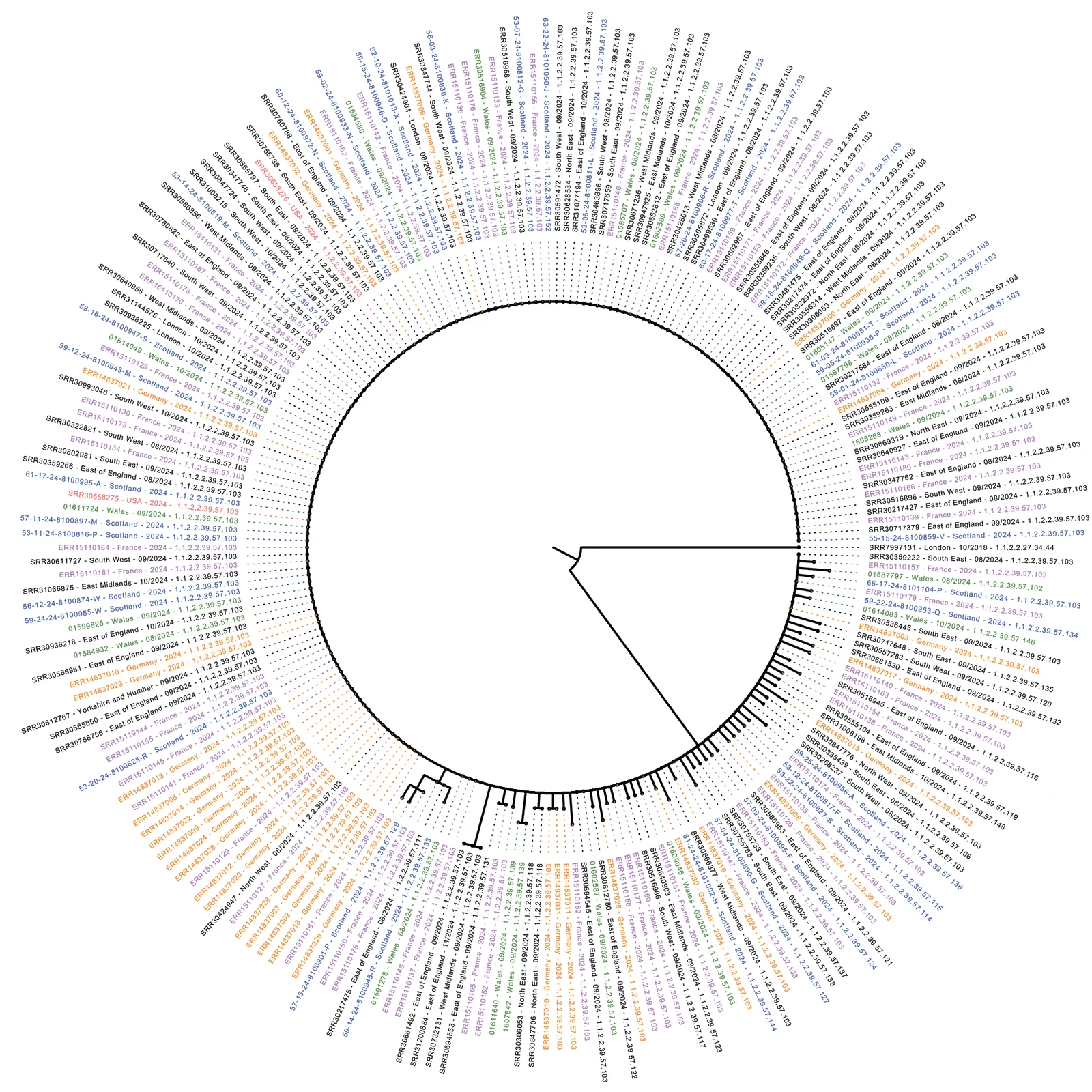
Maximum likelihood phylogeny based of single nucleotide polymorphism alignment of distribution of cases in France, Germany, the United Kingdom and the United States, 2024 (n = 235)

Context isolate (SRR7997131) was used to root the phylogeny.

In the SNP analysis of the *S*. Blockley sequences from cases with travel information and isolates from other countries, we identified two main clusters circulating in the UK: the outbreak described here and a second cluster. This second cluster was identified as HC5_1345, with a SNP address of 1.1.2.2.2.49.%, and with 8–9 alleles difference and an approximate 27 SNPs from the outbreak cluster. In this cluster, there were 53 isolates from the UK, 26 from France and four from the US. Travel to Morocco was reported by 25 UK cases, 17 French cases and two US cases.

## Outbreak response measures

Following a peak in case reporting from the week ending on 25 August 2024, case numbers decreased. The sustained decline coincided with the time frame in which retailers reported a gradual changeover of summer to winter procurement supply chains for fresh produce items. No new cases were reported beyond November 2025, therefore no further public health control measures were considered.

International authorities were notified in accordance with established procedures. The UK FSA authorities notified the retailer of the positive PCR results, and a traceback investigation was initiated. The retailer noted that product with the same use-by-date showed a satisfactory result.

## Discussion

We describe a cross-border investigation into an outbreak of *Salmonella* Blockley in which imported tomatoes were identified as the most likely vehicle of infection. Although not statistically significant, tomatoes were the food item most strongly associated with illness in both univariable and multivariable analyses, with cases who had eaten tomatoes and purchased at Retailer A having higher odds of illness than those who ate tomatoes and shopped at other supermarkets or did not consume tomatoes at all. In the UK, the geographical distribution of cases varied, with the highest case numbers per 100,000 population reported in Scotland, Wales and East of England. The geographical distribution of cases in the UK could reflect distribution networks of imported tomatoes. Food chain traceback investigations identified a common link, with the same grower (Grower X) supplying Retailer A in Scotland and East of England. In parallel, *Salmonella* DNA was detected by PCR from cherry tomatoes, purchased from Retailer A and cultivated by Grower X. Although no single line of evidence was definitive, the cumulative weight of evidence derived from these investigations strengthen the hypothesis that tomatoes were the vehicle for this outbreak.

While the use of a retrospective control group introduces limitations to case–control studies, including questions around the comparability - this option provided a pragmatic, quick and cost-effective way to an applied epidemiological study. A case–case design was not possible, as systematic follow-up of routine salmonellosis cases is not standardised across the UK, leaving no existing dataset with comparable exposure information for rapid analysis. The outbreak investigation was initiated in late August, following an inherent time lag introduced by delays between exposure, symptom onset, sampling and WGS result. This results in challenges in timely response to short shelf-life product outbreak investigations.

The control group was identified from a market-research panel recruited for another investigation of salmonellosis in November 2023. The controls were comparable to cases in age and sex, reducing the likelihood of major structural differences between groups. Although retrospective, we considered that this control group was suitable because tomato consumption patterns are relatively stable across years and seasons. Tomatoes are continuously available in major UK retailers, making major seasonal shifts in consumption patterns unlikely. Previous studies have shown consistent tomato consumption across different food survey methods [10]. In several UK case–control studies on food-borne outbreaks between May to September, tomato consumption in control groups varied from 40 to 45% [14-16], less than the 60% consumption in the control group recruited in November used for this study. We recognise the limitations associated with retrospective control recruitment, however, temporal bias is also a limitation in prospective control recruitment [13], particularly for short shelf-life fresh produce, where suppliers, stock and importers may have changed by the time controls can be recruited and interviewed.

The challenges of under-reporting gastrointestinal infections [17] and the use of varying microbiological techniques (such as WGS) between countries may have underestimated the scale of this outbreak. Vegetables and fruits are consumed in large volumes, and contamination events in global supply chains can affect multiple regions quickly. *Salmonella* surveillance has been significantly enhanced by the adoption of WGS which allows for the rapid identification of related isolates across borders. Also, WGS not only facilitates early detection of outbreaks but enables better understanding of pathogen transmission routes and resistance profiles, crucial for food safety and public health interventions [6].

This cluster of *S.* Blockley had not been identified in the UK before August 2024. Minimal genetic diversity was observed across isolates within the outbreak cluster, indicating a common source for all cases. Analysis of the wider phylogenomic context identified the most closely related *S*. Blockley cluster to the outbreak with an association with travel to Morocco, supportive of an imported food product hypothesis.

In this outbreak, international collaboration and communication through platforms such as EnteroBase and EpiPulse played a pivotal role in the identification and sharing of genomic data. These tools enable quick and efficient communication between public health authorities, aiding in identifying genomic clusters and associated epidemiological and food chain links. Open access data platforms and regulated communication channels have become essential in managing multi-country foodborne outbreaks, where access to real-time data can guide the response and mitigate further cases [6].

Previous outbreaks of *Salmonella* spp. have been documented as associated with tomatoes, including *S.* Senftenberg, *S.* Newport and *S.* Typhimurium, as well as most recently an outbreak of *S.* Strathcona in Europe [18-21]. The findings from this outbreak investigation are therefore consistent with the established biological plausibility for tomatoes as the vehicle [22].

While isolation of *S.* Blockley from tomatoes was not achieved, there are recognised challenges in culture of *Salmonella* from fresh produce. Nonetheless, the potential detection of *Salmonella* spp. by PCR from two imported tomato samples from the same grower as identified through traceback investigations adds weight to the epidemiological and traceback evidence linking these products to the outbreak.

It is possible that contaminated tomatoes were not identified during sampling, as contamination may have been unevenly distributed within batches, with only a small number of individual tomatoes affected. In addition, samples were collected towards the end of the exposure period, which may have further reduced the likelihood of detecting contaminated product. Food testing can be challenging due to low and intermittent contamination levels, sporadic or uneven distribution, low bacterial loads, potentially injured cells and/or inhibited growth conditions, making it harder to isolate pathogens. Although several outbreaks have been caused by fresh produce and tomatoes, detection of *Salmonella* in fresh produce is particularly difficult. Tomatoes are less frequently included in the testing schemes for *Salmonella*; newer assays are frequently adopted to testing meat and products of animal origin [23].

The investigation’s findings were limited by several factors. Although the epidemiological, traceback and microbiological investigations collectively supported imported tomatoes as the most likely vehicle of infection, the analytical epidemiological evidence was not definitive and no culture-confirmed microbiological link to a specific tomato batch was identified. The food chain investigation was further constrained by the challenges of tracing short shelf-life fresh produce items, where supply chains are complex and often lack detailed, batch-specific traceability. For this reason, although the investigation identified a plausible vehicle, it was not possible to determine the specific source of the contamination. Identifying the source and root cause of the outbreak are essential to preventing reoccurrence.

## Conclusion

Findings from this investigation indicate that imported tomatoes from Morocco were the most likely vehicle for this multi-country outbreak of *Salmonella* Blockley. Close collaboration across multiple agencies and international public health agencies allowed integration of epidemiological, microbiological and food chain findings; with the cumulative weight of evidence derived from all components of the investigation substantially strengthening the hypothesis. However, without further investigation into the contamination source, it is possible similar outbreaks may recur. The difficulties of controlling foodborne incidents linked to short, shelf-life fresh produce highlights the need for early detection and an integrated one health approach to mitigate contamination throughout each step of the food chain.

## Data Availability

Representative sequences are available in the Short Read Archive for comparison, accession number SRR30217427; SRR30335439.

## Disclaimer

Marie Chattaway is affiliated to the National Institute for Health Research Health Protection Research Unit (NIHR HPRU) in Gastrointestinal Infections at University of Norwich and Public Health Genomics at University of Birmingham in partnership with UKHSA, in collaboration with University of Newcastle, and are based at UKHSA. The views expressed are those of the author(s) and not necessarily those of the NHS, the NIHR, the Department of Health and Social Care or UKHSA. The findings and conclusions in this report are those of the author(s) and do not necessarily represent the official position of the Centers for Disease Control and Prevention.

## Supplementary Data

649000 Supplementary Material
