# Supplementary material for: A multi-country outbreak of Salmonella Blockley associated with tomato consumption, July to October 2024
Source: Euro Surveill. 2026 Oct 8;31(40):2500862. doi: 10.2807/1560-7917.ES.2026.31.40.2500862 (PMC13653209; doi:10.2807/1560-7917.ES.2026.31.40.2500862)
Supplement: Supplementary file 1 [file 25-00862_HOBAN_Supplement.pdf]

Supplement

This supplementary material is hosted by Eurosurveillance as supporting information alongside the article “A multi-country outbreak of Salmonella Blockley associated with tomato consumption, July to October 2024”, on behalf of the authors, who remain responsible for the accuracy and appropriateness of the content. The same standards for ethics, copyright, attributions and permissions as for the article apply. Supplements are not edited by Eurosurveillance and the journal is not responsible for the maintenance of any links or email addresses provided therein.

Salmonella Blockley ST52 - Aug 24

Questionnaire details

1. Please enter the date on which the questionnaire was completed.*
   dd/mm/yyyy

2. Who is the information in this questionnaire being completed by?*
   Case
   Case's parent
   Other, please specify

Salmonella Blockley ST52 - Aug 24

Case details

Please complete the following details about yourself or the child you're completing the questionnaire for.

3. Please provide your first name.

4. Please provide your last name / surname.

5. Please provide your home address.

6. Please provide your full postcode.

7. Please provide your contact phone numbers.

Home:

Mobile:

8. Please select your sex.

Female
Male
Other

9. Please provide your date of birth.
dd/mm/yyyy

10. Please provide your occupation.

11. Please provide your school or workplace address.

12. Please provide your school or workplace postcode.

Clinical details

Please complete the clinical details about yourself or the child you're completing the questionnaire for.

13. When did you start to feel unwell?

14. Please select the symptoms you experienced.

Diarrhoea *[3 or more loose stools within 24 hrs]*
Blood in stools
Nausea
Vomiting

Abdominal pain
Fever
Headaches
Other, please specify

15. Are you still ill?

Yes
No

How many days were you ill for?

16. Were you admitted to hospital for this illness?

Yes
No

Which hospital were you admitted to?
Date of admission (dd/mm/yy):
Date of discharge (dd/mm/yy):
If exact dates are not known, how many days were you in hospital for?

17. Were you being treated at a clinic or by your GP for any other conditions in the **7 DAYS** before your symptoms first started?

Yes
No

What were you being treated for?

18. Were you taking any of the following in the **7 DAYS** before you became ill?
Please select Yes from the drop-down menus where applicable.

	Yes/No	Type / brand / purchased from
Medicines *[Prescriptions, over the counter, homeopathic medicines]*	-- Please Select --NoYes	
Dietary supplements *[powdered drinks, shakes, tonics]*	-- Please Select --NoYes	
Protein shakes	-- Please Select --NoYes	
Vitamins and minerals *[multivitamins, calcium supplements, herbal remedies]*	-- Please Select --NoYes	

Household details

Please enter the details of the household in which you or the child you're completing the questionnaire for lives.

19. How many people, including yourself, normally live in your household?

20. Did you come into close contact with anyone else with diarrhoea in the **7 DAYS** before you became ill?

Close contact refers to groups such as medical, nursing or care staff in health care settings, teaching staff in nurseries, people caring for ill relatives away from their own household and people in health care institutions or nurseries who might come into contact with other ill patients/pupils.

If YES to Question 20 and you are confident that is this a secondary case, please END THE INTERVIEW.

If in doubt please CONTINUE THE INTERVIEW.

Yes
No

21. Did anyone else in the household have diarrhoea in the **7 DAYS** before you became ill?
Leave blank if no other members of the household experienced illness.

	Names(s)	Age	Sex	Date of onset
Person 1			-- Please Select -- MaleFemale	
Person 2			-- Please Select -- MaleFemale	
Person 3			-- Please Select -- MaleFemale	
Person 4			-- Please Select -- MaleFemale	
Person 5			-- Please Select -- MaleFemale	

Travel history

22. Did you spend any nights outside the UK in the **7 DAYS** before you became ill?
Yes
No

23. If yes to spend any nights outside of the UK in the **7 days** before you became ill, please specify.
Leave all fields blank if no travel outside of the UK.

	Country name	Departure date (dd/mm/yy)	Return date (dd/mm/yy)	Address accommodation
Country 1				
Country 2				
Country 3				
Country 4				
Country 5				

24. Did you spend any nights away from home or travelled but within the UK in the **7 DAYS** before you became ill?
Includes staying at friends or relatives, recreational and business trips etc.

Yes
No

25. If yes to spend any nights away from home or travelled but within the UK in the **7 DAYS** before you became ill, please specify.
Leave all fields blank if no nights were spent away from home.
Places visited: includes hotel in the UK, friend's houses etc.

	Place visited	Town / village	Departure date (dd/mm/yy)	Return date (dd/mm/yy)
Place 1				
Place 2				
Place 3				
Place 4				
Place 5				

26. Did you go on any day trips within the UK in the **7 DAYS** before you became ill?
Includes business visits, work off site, cinemas, shopping trips, gyms, parks etc.

Yes
No

Please give the names and addresses of places visited (include postcodes or area if known)

Eating out/take-aways

27. Did you eat any **meals out at a function** in the **7 DAYS** before you started to feel ill?
Including wedding receptions, birthday, anniversary or dinner parties, clubs, conference dinners, etc.

Yes
No

Please give the name(s) and location(s) of functions attended.

28. Did you eat any food (including take-aways and delivered foods) from or in any of the following places in the **7 DAYS** before you became ill?
Please select Yes from the drop-down menus where applicable.

	Eaten	Details of food/dish	Location of venue
Coffee shop *[eg Starbucks, Costas etc]*	-- Please Select -- NoYes		
Burger bar *[eg McDonalds, Burger King etc]*	-- Please Select -- NoYes		
Pizza parlour *[eg Dominos, Pizza Express etc]*	-- Please Select -- NoYes		
Kebab shop	-- Please Select -- NoYes		
Fish & chip shop	-- Please Select -- NoYes		
Fried chicken bar *[eg KFC]*	-- Please Select -- NoYes		
Bakers shop	-- Please Select -- NoYes		
Sandwich bar	-- Please Select -- NoYes		
Delicatessen *[not in a supermarket]*	-- Please Select -- NoYes		
Vegan/vegetarian restaurant	-- Please Select -- NoYes		
British restaurant	-- Please Select -- NoYes		

Chinese restaurant	-- Please Select -- NoYes
Greek restaurant	-- Please Select -- NoYes
Indian restaurant	-- Please Select -- NoYes
Italian restaurant	-- Please Select -- NoYes
Other restaurant	-- Please Select -- NoYes
Hotel	-- Please Select -- NoYes
Café<br>*[eg greasy spoons, supermarkets etc.]*	-- Please Select -- NoYes
Pub	-- Please Select -- NoYes
Canteen<br>*[eg work, school etc.]*	-- Please Select -- NoYes
Mobile caterer<br>*[eg lunch van, hot dog stand, market stands etc.]*	-- Please Select -- NoYes
Motorway service	-- Please Select -- NoYes
Airport	-- Please Select -- NoYes
Railway station/train	-- Please Select -- NoYes
Petrol station	-- Please Select -- NoYes
Other<br>*[eg ferry, theme park, etc.]*	-- Please Select -- NoYes

Salad, vegetables & herbs

29. Did you eat any of the following **raw** vegetables in the **7 DAYS** before you became ill?
Don't forget vegetables that you've pureed (i.e. in smoothies) or those in sandwiches, burgers, kebabs and as garnishes with takeaway meals etc.
Please select Yes from the drop-down menus where applicable.

	Yes/No	Fresh or frozen	Details, ie. brand, bagged or loose	Purchased from
Mixed salad leaf	-- Please Select -- NoYes	-- Please Select --Not specifiedFreshFrozen		
Iceberg lettuce	-- Please Select -- NoYes	-- Please Select --Not specifiedFreshFrozen		
Little gem	-- Please Select -- NoYes	-- Please Select --Not specifiedFreshFrozen		
Rocket	-- Please Select -- NoYes	-- Please Select --Not specifiedFreshFrozen		
Spinach *[inc. Baby Spinach]*	-- Please Select -- NoYes	-- Please Select --Not specifiedFreshFrozen		
Other type of leaf	-- Please Select -- NoYes	-- Please Select --Not specifiedFreshFrozen		
Other greens *[kale, chard, spring greens]*	-- Please Select -- NoYes	-- Please Select --Not specifiedFreshFrozen		
Salad/Classic tomatoes	-- Please Select -- NoYes	-- Please Select --Not specifiedFreshFrozen		
Cherry tomatoes	-- Please Select -- NoYes	-- Please Select --Not specifiedFreshFrozen		
Plum tomatoes	-- Please Select -- NoYes	-- Please Select --Not specifiedFreshFrozen		

Vine tomatoes	-- Please Select -- NoYes	-- Please Select --Not specifiedFreshFrozen
Other tomato	-- Please Select -- NoYes	-- Please Select --Not specifiedFreshFrozen
Whole cucumber	-- Please Select -- NoYes	-- Please Select --Not specifiedFreshFrozen
Cucumber portions	-- Please Select -- NoYes	-- Please Select --Not specifiedFreshFrozen
Avocado	-- Please Select -- NoYes	-- Please Select --Not specifiedFreshFrozen
Spring onions	-- Please Select -- NoYes	-- Please Select --Not specifiedFreshFrozen
Red Peppers	-- Please Select -- NoYes	-- Please Select --Not specifiedFreshFrozen
Yellow Peppers	-- Please Select -- NoYes	-- Please Select --Not specifiedFreshFrozen
Orange Peppers	-- Please Select -- NoYes	-- Please Select --Not specifiedFreshFrozen
Green Peppers	-- Please Select -- NoYes	-- Please Select --Not specifiedFreshFrozen
Olives	-- Please Select -- NoYes	-- Please Select --Not specifiedFreshFrozen
Sprouted seeds *[inc. alfafa, bean sprouts etc]*	-- Please Select -- NoYes	-- Please Select --Not specifiedFreshFrozen
Brussel sprouts	-- Please Select -- NoYes	-- Please Select --Not specifiedFreshFrozen
Onions	-- Please Select -- NoYes	-- Please Select --Not specifiedFreshFrozen
Cabbage	-- Please Select -- NoYes	-- Please Select --Not specifiedFreshFrozen

Mushrooms<br>*[inc. shiitake, porcini, any other shrooms]*	-- Please Select -- NoYes	-- Please Select --Not specifiedFreshFrozen
Beetroot	-- Please Select -- NoYes	-- Please Select --Not specifiedFreshFrozen
Sweetcorn	-- Please Select -- NoYes	-- Please Select --Not specifiedFreshFrozen
Baby corn	-- Please Select -- NoYes	-- Please Select --Not specifiedFreshFrozen
Green beans	-- Please Select -- NoYes	-- Please Select --Not specifiedFreshFrozen
Broad beans	-- Please Select -- NoYes	-- Please Select --Not specifiedFreshFrozen
Mange tout	-- Please Select -- NoYes	-- Please Select --Not specifiedFreshFrozen
Runner beans	-- Please Select -- NoYes	-- Please Select --Not specifiedFreshFrozen
Sugar snap peas	-- Please Select -- NoYes	-- Please Select --Not specifiedFreshFrozen
Celery	-- Please Select -- NoYes	-- Please Select --Not specifiedFreshFrozen
Peas	-- Please Select -- NoYes	-- Please Select --Not specifiedFreshFrozen
Broccoli	-- Please Select -- NoYes	-- Please Select --Not specifiedFreshFrozen
Tenderstem broccoli	-- Please Select -- NoYes	-- Please Select --Not specifiedFreshFrozen
Cauliflower	-- Please Select -- NoYes	-- Please Select --Not specifiedFreshFrozen
Carrots	-- Please Select -- NoYes	-- Please Select --Not specifiedFreshFrozen

Asparagus	-- Please Select -- NoYes	-- Please Select --Not specifiedFreshFrozen
Aubergine	-- Please Select -- NoYes	-- Please Select --Not specifiedFreshFrozen
Other vegetables	-- Please Select -- NoYes	-- Please Select --Not specifiedFreshFrozen

30. Did you eat any of the following **ready-made pre-packed salads** in the **7 DAYS** before you became ill?
Please select Yes from the drop-down menus where applicable.
*If selecting **Yes** for **Other**, please specify in the free-text box.*

	Eaten	Details, ie. brand/products in salad	Purchased from
Italian style salad	-- Please Select -- NoYes		
Tomato and mozzarella salad bowl	-- Please Select -- NoYes		
Moroccan style	-- Please Select -- NoYes		
Lentil salad	-- Please Select -- NoYes		
Bulgar wheat salad	-- Please Select -- NoYes		
Potato salad	-- Please Select -- NoYes		
Quinoa/grains salad	-- Please Select -- NoYes		
Wild rice salad bowl	-- Please Select -- NoYes		
Other	-- Please Select -- NoYes		

31. Did you eat any of the following **herbs** in the **7 DAYS** before you became ill?
Don't forget vegetables that you've pureed (i.e. in smoothies) or those in sandwiches, burgers, kebabs and as garnishes with takeaway meals etc.
Please select Yes from the drop-down menus where applicable.

	Fresh	Dried	Details, ie. type, brand	Purchased from
Mint	-- Please Select -- NoYes	-- Please Select -- NoYes		
Basil	-- Please Select -- NoYes	-- Please Select -- NoYes		
Coriander	-- Please Select -- NoYes	-- Please Select -- NoYes		
Parsley	-- Please Select -- NoYes	-- Please Select -- NoYes		
Thyme	-- Please Select -- NoYes	-- Please Select -- NoYes		
Chives	-- Please Select -- NoYes	-- Please Select -- NoYes		
Dill	-- Please Select -- NoYes	-- Please Select -- NoYes		
Rosemary	-- Please Select -- NoYes	-- Please Select -- NoYes		
Lemongrass	-- Please Select -- NoYes	-- Please Select -- NoYes		
Others	-- Please Select -- NoYes	-- Please Select -- NoYes		

32. Did you eat any of the following **spices** in the **7 DAYS** before you became ill?
Don't forget vegetables that you've pureed (i.e. in smoothies) or those in sandwiches, burgers, kebabs and as garnishes with takeaway meals etc.
Please select Yes from the drop-down menus where applicable.

	Fresh	Dried	Details, ie. type, brand	Purchased from
Pepper	-- Please Select -- NoYes	-- Please Select -- NoYes		
Ginger	-- Please Select -- NoYes	-- Please Select -- NoYes		

Chilli	-- Please Select -- NoYes	-- Please Select -- NoYes
Cumin	-- Please Select -- NoYes	-- Please Select -- NoYes
Cinnamon	-- Please Select -- NoYes	-- Please Select -- NoYes
Paprika	-- Please Select -- NoYes	-- Please Select -- NoYes
Tumeric	-- Please Select -- NoYes	-- Please Select -- NoYes
Cardamom	-- Please Select -- NoYes	-- Please Select -- NoYes
Other	-- Please Select -- NoYes	-- Please Select -- NoYes

Fruit

33. Did you eat any of the following **fresh fruit** in the **7 DAYS** before you became ill?
Please select Yes from the drop-down menus where applicable.
*Ensure to ask about frozen products i.e. in smoothie mixes etc also. If selecting **Yes** for **Other**, please specify in the free-text box.*

	Yes/No	Fresh/Frozen	Details	Purchased from
Apples	-- Please Select -- NoYes	-- Please Select -- Fresh/chilledFrozen		
Pears	-- Please Select -- NoYes	-- Please Select -- Fresh/chilledFrozen		
Bananas	-- Please Select -- NoYes	-- Please Select -- Fresh/chilledFrozen		

Grapes	-- Please Select -- NoYes	-- Please Select -- Fresh/chilledFrozen
Oranges	-- Please Select -- NoYes	-- Please Select -- Fresh/chilledFrozen
Clementines	-- Please Select -- NoYes	-- Please Select -- Fresh/chilledFrozen
Satsumas	-- Please Select -- NoYes	-- Please Select -- Fresh/chilledFrozen
Tangerines	-- Please Select -- NoYes	-- Please Select -- Fresh/chilledFrozen
Lemons	-- Please Select -- NoYes	-- Please Select -- Fresh/chilledFrozen
Peaches	-- Please Select -- NoYes	-- Please Select -- Fresh/chilledFrozen
Nectarines	-- Please Select -- NoYes	-- Please Select -- Fresh/chilledFrozen
Apricots	-- Please Select -- NoYes	-- Please Select -- Fresh/chilledFrozen
Plums	-- Please Select -- NoYes	-- Please Select -- Fresh/chilledFrozen
Strawberries	-- Please Select -- NoYes	-- Please Select -- Fresh/chilledFrozen
Raspberries	-- Please Select -- NoYes	-- Please Select -- Fresh/chilledFrozen
Blueberries	-- Please Select -- NoYes	-- Please Select -- Fresh/chilledFrozen
Cranberries	-- Please Select -- NoYes	-- Please Select -- Fresh/chilledFrozen
Blackberries	-- Please Select -- NoYes	-- Please Select -- Fresh/chilledFrozen

Watermelon	-- Please Select -- NoYes	-- Please Select -- Fresh/chilledFrozen		
Cherries	-- Please Select -- NoYes	-- Please Select -- Fresh/chilledFrozen		
Galia melon	-- Please Select -- NoYes	-- Please Select -- Fresh/chilledFrozen		
Cantaloupe melon	-- Please Select -- NoYes	-- Please Select -- Fresh/chilledFrozen		
Honeydew melon	-- Please Select -- NoYes	-- Please Select -- Fresh/chilledFrozen		
Other melon	-- Please Select -- NoYes	-- Please Select -- Fresh/chilledFrozen		
Pomegranate	-- Please Select -- NoYes	-- Please Select -- Fresh/chilledFrozen		
Mango	-- Please Select -- NoYes	-- Please Select -- Fresh/chilledFrozen		
Pineapple	-- Please Select -- NoYes	-- Please Select -- Fresh/chilledFrozen		
Other fruits *[eg. lychees, etc]*	-- Please Select -- NoYes	-- Please Select -- Fresh/chilledFrozen		

34. Did you eat any **prepacked fruit mixes** in the **7 DAYS** before you became ill?
Please select Yes from the drop-down menus where applicable.
*If selecting **Yes** for **Other**, please specify in the free-text box.*

	Yes/No	Fresh/Frozen	Details	Purchased from
Summer fruit mix	-- Please Select -- NoYes	-- Please Select -- Fresh/chilledFrozen		
Tropical fruit mix	-- Please Select -- NoYes	-- Please Select -- Fresh/chilledFrozen		
Smoothie fruit mix	-- Please Select -- NoYes	-- Please Select -- Fresh/chilledFrozen		

Pre-cut fruit salad bowls	-- Please Select -- NoYes	-- Please Select -- Fresh/chilledFrozen
Other fruit mix	-- Please Select -- NoYes	-- Please Select -- Fresh/chilledFrozen

35. Did you eat any of the following **dried fruits, nuts or seeds** in the **7 DAYS** before you became ill?
Don't forget vegetables that you've pureed (i.e. in smoothies) or those in sandwiches, burgers, kebabs and as garnishes with takeaway meals etc.
Please select Yes from the drop-down menus where applicable.

	**Yes / No**	**Details, ie. brand name etc**	**Purchased from**
Raisins/sultanas	-- Please Select -- NoYes		
Dates	-- Please Select -- NoYes		
Coconut	-- Please Select -- NoYes		
Mixed dried fruit	-- Please Select -- NoYes		
Almonds	-- Please Select -- NoYes		
Pistachios	-- Please Select -- NoYes		
Walnuts	-- Please Select -- NoYes		
Hazelnuts	-- Please Select -- NoYes		
Cashews	-- Please Select -- NoYes		
Peanuts	-- Please Select -- NoYes		
Brazil nuts	-- Please Select -- NoYes		

Pine nuts	-- Please Select -- NoYes
Mixed nuts	-- Please Select -- NoYes
Sesame	-- Please Select -- NoYes
Sunflower	-- Please Select -- NoYes
Chia	-- Please Select -- NoYes
Pumpkin	-- Please Select -- NoYes
Flax	-- Please Select -- NoYes
Other seeds	-- Please Select -- NoYes
Other mixed fruit/nut/seed	-- Please Select -- NoYes

36. Did you eat any of the following **products** made with **nuts and seeds** in the **7 DAYS** before you became ill?
Please select 'Yes' from the drop-down menus where applicable.
*If selecting **Yes** to **Other**, please specify in the free-text box.*

	Eaten	Details, ie. type, brand	Purchased from
Tahini	-- Please Select -- NoYes		
Hummus	-- Please Select -- NoYes		
Baklava	-- Please Select -- NoYes		
Other	-- Please Select -- NoYes		

Poultry

37. Did you eat any of the following foods in the **7 DAYS** before you became ill?
Please select 'Yes' from the drop-down menus where applicable.
*If selecting **Yes** to **Other poultry**, please specify in the free-text box.*

	Eaten	Details, ie. dishes / brand	Purchased from
Whole chicken	-- Please Select -- NoYes		
Chicken breast	-- Please Select -- NoYes		
Diced chicken pieces	-- Please Select -- NoYes		
Chicken drumsticks	-- Please Select -- NoYes		
Chicken thigh portions	-- Please Select -- NoYes		
Chicken legs	-- Please Select -- NoYes		
Chicken wings	-- Please Select -- NoYes		
Minced chicken	-- Please Select -- NoYes		
Chicken offal	-- Please Select -- NoYes		
Chicken liver pâté/parfait	-- Please Select -- NoYes		
Breaded chicken fillets	-- Please Select -- NoYes		

Fried/battered chicken	-- Please Select -- NoYes
Chicken pies/pastries	-- Please Select -- NoYes
Chicken burgers	-- Please Select -- NoYes
Chicken nuggets/goujons	-- Please Select -- NoYes
Chicken stir fry	-- Please Select -- NoYes
Chicken fried rice	-- Please Select -- NoYes
Chicken curry	-- Please Select -- NoYes
Chicken sausages	-- Please Select -- NoYes
Chicken slices/sandwich meat	-- Please Select -- NoYes
Chicken soups/stews	-- Please Select -- NoYes
Other prepared chicken dishes *[eg. hunters chicken, chicken fajita kits, etc]*	-- Please Select -- NoYes
Whole turkey	-- Please Select -- NoYes
Turkey portions	-- Please Select -- NoYes
Whole duck	-- Please Select -- NoYes
Duck portions	-- Please Select -- NoYes

Other poultry or game birds *[eg goose, pheasant, guinea fowl, quail, partridge etc]* *Please specify*	-- Please Select -- NoYes

38. Was any **raw poultry** handled in your household in the 7 days before your illness onset?

Yes
No

39. Did you handle **raw poultry** in the 7 days before onset?

Yes
No

Beef

40. Did you eat any of the following foods containing beef in the **7 DAYS** before you became ill?
Please select 'Yes' from the drop-down menus where applicable.
*If selecting **Yes** for **Other beef**, please specify in the free-text box.*

	Eaten	Details, ie. dishes / brand	Purchased from
Burgers	-- Please Select -- NoYes		
Minced beef dishes *[Bolognaise, lasagne, chilli, meatballs etc]*	-- Please Select -- NoYes		
Steak	-- Please Select -- NoYes		
Roast beef *[joints etc]*	-- Please Select -- NoYes		
Beef stews/casseroles/curries	-- Please Select -- NoYes		
Pies and pasties *[Steak & kidney pies, Cornish pasties etc]*	-- Please Select -- NoYes		

Cold roast/corned beef	-- Please Select -- NoYes
Beef sausages *[Including hot dogs]*	-- Please Select -- NoYes
Other beef *Please specify*	-- Please Select -- NoYes

41. Was any **raw beef** handled in your household in the **7 DAYS** before you became ill?
Including joints, steaks, mince, burgers, etc.

Yes
No

42. Did <u>you</u> handle **raw beef** in the 7 days before onset?
Including joints, steaks, mince, burgers, etc.

Yes
No

Pork

43. Did you eat any of the following foods containing pork in the **7 DAYS** before you became ill?
Please select 'Yes' from the drop-down menus where applicable.
*If selecting **Yes** for **Other**, please specify in the free-text box.*

	Eaten	Details, ie. dishes / brand	Purchased from
Pork sausages	-- Please Select -- NoYes		
Bacon	-- Please Select -- NoYes		
Gammon	-- Please Select -- NoYes		

Pork chops	-- Please Select -- NoYes
Pork casseroles/strew	-- Please Select -- NoYes
Pork mince	-- Please Select -- NoYes
Roast pork	-- Please Select -- NoYes
Pork pies	-- Please Select -- NoYes
Pâté	-- Please Select -- NoYes
Salami	-- Please Select -- NoYes
Pepperoni	-- Please Select -- NoYes
Ham *[Not including Parma, Serrano ham etc]*	-- Please Select -- NoYes
Dry cured ham *[eg Parma, Serrano, Bayonne ham etc]*	-- Please Select -- NoYes
Luncheon meat	-- Please Select -- NoYes
Sausage rolls	-- Please Select -- NoYes
Scotch eggs	-- Please Select -- NoYes
Other pork dishes [e.g. stir fries, etc] *Please specify*	-- Please Select -- NoYes

44. Was any **raw pork** handled in your household in the **7 DAYS** before you became ill?
Including joints, chops, mince, sausages.

Yes
No

45. Did you handle **raw pork** in the 7 days before onset?
Including joints, chops, mince, sausages.

Yes
No

Other meat and fish products

46. Did you eat any of the following foods in the **7 DAYS** before you became ill?
Please select Yes from the drop-down menus where applicable.
*If selecting **Yes** for **exotic meat** or **Other**, please specify in the free-text box(es).*

	Eaten	Details (e.g. dishes/brand)	Purchased from
Kebabs	-- Please Select -- NoYes		
Pizza with meat *[eg pepperoni etc]*	-- Please Select -- NoYes		
Other *[eg Exotic/game meat etc]*	-- Please Select -- NoYes		

47. Did you eat any of the following **fish or shellfish products** in the **7 DAYS** before you became ill?
Please select Yes from the drop-down menus where applicable.
*If selecting **Yes** for **Other**, please specify in the free-text box.*

	Yes/No	Fresh/Frozen	Details	Purchased from
White fish	-- Please Select -- NoYes	-- Please Select -- Fresh/chilledFrozen		
Salmon	-- Please Select -- NoYes	-- Please Select -- Fresh/chilledFrozen		
Tuna	-- Please Select -- NoYes	-- Please Select -- Fresh/chilledFrozen		
Other finfish	-- Please Select -- NoYes	-- Please Select -- Fresh/chilledFrozen		

Prawns	-- Please Select -- NoYes	-- Please Select -- Fresh/chilledFrozen
Other shellfish	-- Please Select -- NoYes	-- Please Select -- Fresh/chilledFrozen

Salmonella Blockley ST52 - Aug 24

Eggs and vegetarian products

48. Did you eat any of the following foods in the **7 DAYS** before you became ill?

	Yes/No	Details	Purchased from
Eggs	-- Please Select -- NoYes		
Omelettes/scrambled eggs	-- Please Select -- NoYes		
Egg sandwiches/salads	-- Please Select -- NoYes		
Quiches	-- Please Select -- NoYes		
Mayonnaise	-- Please Select -- NoYes		
Custard products	-- Please Select -- NoYes		
Other egg products	-- Please Select -- NoYes		

49. Did you eat any of the following **vegetarian foods** in the **7 DAYS** before you became ill?

	Yes/No	Brand/details	Purchased from

Tofu/tofu products	-- Please Select -- NoYes
Bean curd/bean curd products	-- Please Select -- NoYes
Quorn/quorn product	-- Please Select -- NoYes
Veggie burgers/sausages	-- Please Select -- NoYes
Paneer	-- Please Select -- NoYes
Soya based products	-- Please Select -- NoYes
Other bean based products	-- Please Select -- NoYes
Other	-- Please Select -- NoYes

Sandwiches

50. Did you eat any **sandwiches, rolls, filled baguettes** or **wraps** that were **bought or served away from home** in the **7 DAYS** before you became ill?
Includes prepacked sandwiches from shops/railway stations, works/school canteens, rolls at buffet lunches, custom made sandwiches from sandwich bars, pubs etc.

Yes
No

51. If **Yes**, did you eat any of the following types of sandwich?
Please select Yes from the drop-down menus where applicable.
*If selecting **Yes** for **other**, please specify in the free-text box.*

Eaten	Details	Purchased from

Ham	-- Please Select -- NoYes
Beef *[eg roast beef, corned beef, pastrami etc]*	-- Please Select -- NoYes
Bacon/BLT	-- Please Select -- NoYes
Chicken	-- Please Select -- NoYes
Turkey	-- Please Select -- NoYes
Other meat *[eg salami, sausage etc]*	-- Please Select -- NoYes
Tuna	-- Please Select -- NoYes
Salmon *[Incl. smoked salmon]*	-- Please Select -- NoYes
Prawn / other seafood *[eg crab, crayfish, etc.]*	-- Please Select -- NoYes
Egg mayonnaise	-- Please Select -- NoYes
Other egg	-- Please Select -- NoYes
Cheese	-- Please Select -- NoYes
Plant based/vegetarian	-- Please Select -- NoYes
Other *Please specify*	-- Please Select -- NoYes

52. Did any of these sandwiches include any of the following extras?
Please select Yes from the drop-down menus where applicable.
*If selecting **Yes** for **other**, please specify in the free-text box.*

	Eaten	Details	Purchased from
Cucumber	-- Please Select -- NoYes		
Lettuce	-- Please Select -- NoYes		
Tomato	-- Please Select -- NoYes		
Spinach	-- Please Select -- NoYes		
Onions	-- Please Select -- NoYes		
Cress	-- Please Select -- NoYes		
Other leaves	-- Please Select -- NoYes		

Snacks and dairy products

53. Did you eat any of the following **snack products** in the **7 DAYS** before you became ill?
Please select 'Yes' from the drop-down menus where applicable.
*If selecting **Yes** to **Other**, please specify in the free-text box.*

	Eaten	Details, ie. type, brand	Purchased from
Biscuits	-- Please Select -- NoYes		
Cereals	-- Please Select -- NoYes		
Pastries	-- Please Select -- NoYes		

Cakes	-- Please Select -- NoYes
Crisps	-- Please Select -- NoYes
Crackers	-- Please Select -- NoYes
Confectionery	-- Please Select -- NoYes
Chocolate	-- Please Select -- NoYes
Other	-- Please Select -- NoYes

54. Did you eat any of the following **types of milk products** in the **7 DAYS** before you became ill?
Please select 'Yes' from the drop-down menus where applicable.
*If selecting **Yes** to **Other**, please specify in the free-text box.*

	**Eaten**	**Details, ie. type, brand**	**Purchased from**
Dairy milk	-- Please Select -- NoYes		
Oat milk	-- Please Select -- NoYes		
Almond milk	-- Please Select -- NoYes		
Soy milk	-- Please Select -- NoYes		
Coconut milk	-- Please Select -- NoYes		
Other milk based product	-- Please Select -- NoYes		

55. Did you have any **cheese** in the **7 days** before becoming unwell?

**Yes/No**	**Details/brand**

Hard cheese	-- Please Select -- NoYes		
Soft cheese	-- Please Select -- NoYes		

56. Did you have any **other dairy products** in the **7 DAYS** before your illness onset?

	Yes/No	Details	Purchased from
Other dairy	-- Please Select -- NoYes		

Shops

57. Have you bought any food from the following shops recently?
Including bread, chicken, meat, milk, vegetables, processed foods, etc.

	Shopped at	Name / branch / location
Aldi	-- Please Select -- NoYes	
Asda	-- Please Select -- NoYes	
Budgens	-- Please Select -- NoYes	
Co-op	-- Please Select -- NoYes	
Iceland	-- Please Select -- NoYes	
Lidl	-- Please Select -- NoYes	

Marks & Spencer	-- Please Select -- NoYes
Morrisons	-- Please Select -- NoYes
Netto	-- Please Select -- NoYes
Sainsbury	-- Please Select -- NoYes
Somerfield	-- Please Select -- NoYes
Spar	-- Please Select -- NoYes
Tesco	-- Please Select -- NoYes
Waitrose	-- Please Select -- NoYes

58. Have you eaten any food that was **bought abroad** in the **7 DAYS** before you were ill?
Bought by yourself or given to you as a gift.

Yes
No

Please specify the type of food(s) and country of purchase.

Contact with animals & natural environment

59. Have you come into contact with any animals at home or outside your home?

Dog(s)
Cat(s)
Fish
Bird(s) *[eg budgies, parrots, etc]*
Reptile(s) *[eg snakes, lizards, tortoises, etc]*
Rodents*hamsters,mice*

Chickens
Cattle
Horses
Pigs
Sheep/lambs
Other, please specify *[eg rabbits, frogs, etc]*

60. If yes: please give details on the brand and type of **PET FOOD** that you use, and where you normally buy it.
Fill in rows as appropriate. There is space provided for 3 of each type of food

	**Animal**	**Brand**	**Where purchased**
Dry food	-- Please Select -- DogCatFishBirdReptileRodentOther		
Wet food	-- Please Select -- DogCatFishBirdReptileRodentOther		
Other food/treats	-- Please Select -- DogCatFishBirdReptileRodentOther		

61. Do you feed your pet raw meat pet food? If so, please give details:
Fill in rows as appropriate.

	**Yes/No**	**Brand/Type**	**Where purchased**
Raw meat - prepared at home	-- Please Select -- NoYes		
Raw pet food - commercially made	-- Please Select -- NoYes		

62. Had you undertaken any gardening in the 7 days prior to onset? Or had contact with wildlife/soil etc outdoors [i.e. by walking outside in fields, farms etc]?

	**Yes/No**	**Details**
Gardening	-- Please Select -- NoYes	
Wildlife/outdoors exposure	-- Please Select -- NoYes	
Other	-- Please Select -- NoYes	

63. If yes to gardening, please give details of any products used:

	**Yes/No**	**Brand name**	**Purchase location**

Compost	-- Please Select -- NoYes
Chemical fertiliser *[i.e. Nitrogen, phosphorus, potassium etc]*	-- Please Select -- NoYes
Bone meal fertiliser	-- Please Select -- NoYes
Manure	-- Please Select -- NoYes
Chicken litter manure	-- Please Select -- NoYes
Fruit/vegetable feed	-- Please Select -- NoYes
Other product	-- Please Select -- NoYes

Loyalty card data

Note to interviewer: Please read the text directly as written when speaking to the case.

64. Do you hold one or more loyalty cards for the supermarkets you regularly shop at?

    Yes
    No

65. If **YES:** Would you be willing to provide the name of the shop(s), name on the card and the loyalty card number(s) to enable the public health protection authorities and food standards agency to contact the retailer to request more details of particular foods of relevence to our investigation that you have purchased from them, for example brand, item, date of purchase, packet size, best before date, batch number etc? This information will be shared **ONLY** between relevant public health and food safety services and the retailer and used to aid our investigation.

Please note - by agreeing to this and providing your card details you are indicating informed consent to this request

Yes, I consent to providing my loyalty card data to be used in the manner described:

Yes
No

66. Card 1 (shop, number and name on card)

67. Card 2 (shop, number and name on card)

68. Card 3 (shop, number and name on card)

End of questionnaire

69. **Thank you for your co-operation**

If required, would it be possible for us to contact you again if additional information is needed?

Yes
No
